# Early life stress and the pathogenesis of visceral hypersensitivity: mechanisms and implications for disorders of gut-brain interaction

**DOI:** 10.3389/fcell.2026.1827148

**Published:** 2026-06-05

**Authors:** Enfu Tao

**Affiliations:** Department of Neonatology and NICU, Wenling Maternal and Child Healthcare Hospital, Wenling, Zhejiang, China

**Keywords:** brain-gut axis, early life stress, epigenetics, irritable bowel syndrome, neuroinflammation, sex differences, therapeutic targets, visceral hypersensitivity

## Abstract

Early life stress (ELS) is a major risk factor for disorders of gut-brain interaction (DGBIs), particularly irritable bowel syndrome (IBS), through the induction of persistent visceral hypersensitivity. This review synthesizes evidence from epidemiological studies, clinical observations, and preclinical models to establish ELS-induced visceral hypersensitivity as a core pathophysiological axis. I delineate the multi-level mechanisms underlying this phenomenon, encompassing: (1) central neuroendocrine dysregulation (hypothalamic-pituitary-adrenal axis, corticotropin-releasing factor signaling) with epigenetic modifications (histone acetylation, non-coding RNAs) in limbic circuits; (2) spinal sensitization via brain-derived neurotrophic factor upregulation, glutamate transporter dysfunction, and potassium channel downregulation; (3) peripheral alterations including enterochromaffin cell hyperplasia, mast cell activation, enteric glial phenotypic switching, barrier disruption, and microbiota dysbiosis. I highlight critical modifiers including sexual dimorphism (estrogen-dependent mechanisms) and resilience factors (benevolent childhood experiences, environmental enrichment). Finally, I present a translational roadmap integrating pharmacological, nutritional, and behavioral interventions targeting ELS-programmed dysfunction along the brain-gut axis, emphasizing opportunities for prevention and precision medicine.

## 1 Introduction

Disorders of gut-brain interaction (DGBIs), most notably irritable bowel syndrome (IBS), impose a considerable global burden on patients and healthcare systems, primarily driven by chronic visceral pain and discomfort (Woods et al., 2026). These functional conditions are defined by symptom-based criteria in the absence of detectable structural or biochemical abnormalities, a characteristic that has historically complicated both their conceptualization and treatment (Talley et al., 2015; Mayer and Collins, 2002). Contemporary understanding has firmly established DGBIs within a biopsychosocial framework, wherein symptoms arise from complex, bidirectional dysregulation along the brain-gut axis (Woods et al., 2026; Almounajed and Drossman, 1996). Within this paradigm, visceral hypersensitivity (VH)—a state of heightened perception and lowered threshold to pain from gut stimuli—has emerged as a cardinal pathophysiological mechanism underpinning the hallmark symptom of chronic abdominal pain (Mayer and Collins, 2002; Liu et al., 2017). This sensory aberration is not an isolated phenomenon but is intricately linked to a spectrum of psychosocial comorbidities, particularly anxiety and depression, suggesting shared neural and molecular substrates (Greenwood-Van and Johnson, 2017; Liu, 2025).

A critical environmental factor consistently implicated in the vulnerability to develop both visceral pain disorders and associated psychiatric conditions is exposure to adverse experiences during early life (Jones, 2016; Burke et al., 2017; Wu, 2012). Epidemiological and clinical studies robustly demonstrate that a history of early life stress (ELS), such as trauma, neglect, or abuse, significantly increases the risk for the later development of IBS and other functional gastrointestinal disorders (FGIDs) (Fuentes and Christianson, 2018; Levy et al., 2006; Al-Beltagi et al., 2025; Dendrinos et al., 2025). This association is not merely correlative; ELS is thought to facilitate maladaptive, long-lasting changes in the neurobiological systems that govern stress responsivity, emotional regulation, and pain processing (Burke et al., 2017; Wu, 2012). The convergence of these alterations is thought to promote a persistent state of central sensitization, which may lower the threshold for visceral pain perception and is associated with amplified gastrointestinal symptoms in response to subsequent physiological or psychological stressors throughout life (Meerveld and Johnson, 2018; Lovick, 2016). The high comorbidity between DGBIs and stress-related psychiatric disorders further supports the concept of a common origin rooted in early adversity (Liu, 2025; O’Mahony et al., 2017).

The mechanisms by which ELS may translates into a lifelong predisposition for VH are thought to involve a multifaceted disruption of the brain-gut axis (O’Mahony et al., 2017; Buckley et al., 2014). At the core is a dysregulated stress response system, frequently manifested as alterations in hypothalamic-pituitary-adrenal (HPA) axis function and corticotropin-releasing factor (CRF) signaling (Burke et al., 2017; Meerveld and Johnson, 2018; Kehne, 2007). CRF, acting through its receptors, serves as a master coordinator of the stress response, and its hyperactivation is linked to both anxiety and visceral pain states (Kehne, 2007; Kehne and Cain, 2010). ELS can permanently alter the set-point of this system, leading to aberrant glucocorticoid signaling and enhanced central drive on peripheral gut function (Fuentes and Christianson, 2018; Chaloner and Greenwood-Van, 2013a). This neuro-endocrine dysregulation converges with immune and neural pathways. It influences gut barrier integrity, mucosal immunity, enteric nervous system (ENS) activity, and gut microbiota composition (Al-Beltagi et al., 2025; Buckley et al., 2014). The resulting state of low-grade immune activation and altered gut microenvironment can, in turn, send afferent signals back to the central nervous system, perpetuating a cycle of hypersensitivity and symptom generation (Liu, 2025; Wu, 2012).

Crucially, the effects of ELS exhibit significant sexual dimorphism, with women being disproportionately affected by both DGBIs and stress-related psychiatric disorders (Chaloner and Greenwood-Van, 2013a; Prusator et al., 2016). This disparity underscores the importance of considering sex hormones as key modulators of the stress-pain axis. Preclinical animal models have been indispensable in recapitulating the long-term consequences of ELS on visceral sensitivity and in disentangling these complex, interacting mechanisms (Mayer and Collins, 2002; Fuentes and Christianson, 2018; Prusator et al., 2016). These models confirm that adverse early experiences can produce enduring, often sex-specific, increases in visceral pain responses in adulthood, mirroring clinical observations (Greenwood-Van and Johnson, 2017; Chaloner and Greenwood-Van, 2013a). Furthermore, emerging evidence points to epigenetic modifications as a primary molecular interface, providing a plausible mechanism for how transient environmental exposures during critical developmental windows can engrain lasting changes in gene expression within neural and peripheral circuits, thereby encoding vulnerability for future disease (Liu et al., 2017; Théodorou, 2013).

This review aims to synthesize the substantial body of evidence, from epidemiological associations to molecular insights, that positions ELS and VH as a core pathophysiological axis in DGBIs. It will explore the clinical and preclinical data linking ELS to adult visceral pain, dissect the involved neuro-endocrine-immune pathways from the brain to the gut, and examine the epigenetic mechanisms that underpin this enduring vulnerability. By integrating these perspectives, the review underscores the necessity of a holistic, biopsychosocial approach to patient care—one that acknowledges the legacy of early adversity and targets the dysregulated brain-gut axis with integrated pharmacological, psychological, and dietary strategies (Woods et al., 2026; Liu, 2025; Dendrinos et al., 2025).

## 2 Epidemiological and clinical evidence linking early life adversity to adult visceral hypersensitivity and irritable bowel syndrome

A substantial body of epidemiological and clinical research provides compelling evidence for the association between adverse experiences in early life and the subsequent development of IBS and its hallmark feature, VH. This relationship is a cornerstone of the biopsychosocial model of IBS, highlighting how environmental factors, particularly during critical developmental windows, can program long-term vulnerability within the brain-gut axis (Woods et al., 2026; Talley et al., 2015). Systematic reviews and meta-analyses consistently demonstrate that a history of adverse childhood experiences (ACEs), encompassing abuse, neglect, and household dysfunction, significantly increases the odds of developing IBS in adulthood (Joshee et al., 2022; Lenover et al., 2025). The pooled evidence indicates that individuals with a history of any childhood trauma event have approximately 1.6 times greater odds of having IBS compared to those without such a history, with physical and sexual trauma emerging as particularly strong predictors (Lenover et al., 2025; Halland et al., 2014). The association appears robust across different populations and cultures, as studies from North America, Europe, the Middle East, and Latin America report a higher prevalence of ACEs among IBS patients, ranging from 63% to 80%, compared to 48%–59% in healthy controls (Halland et al., 2014; Alsubaie et al., 2022; Priego-Parra et al., 2024; Berens et al., 2020).

The nature and perception of the traumatic event are critical modifiers of risk. Beyond the mere presence of an ACE, the perceived severity of the trauma and the emotional response at the time, particularly the experience of fear, are independently associated with increased odds of IBS (Ju et al., 2020; Rahal et al., 2020). Furthermore, the relationship often exhibits a dose-response pattern, where a greater number of ACEs correlates with increased risk for IBS and greater gastrointestinal symptom severity (Alsubaie et al., 2022; Priego-Parra et al., 2024; Bryan and Beitz, 2021; Park et al., 2016). For instance, the likelihood of having IBS increases with each additional ACE, and patients with severe IBS symptoms are more likely to report exposure to four or more ACEs (Priego-Parra et al., 2024; Park et al., 2016). This graded relationship underscores the cumulative burden of ELS on gut-brain homeostasis. Importantly, some evidence suggests protective factors may mitigate this risk. Emotional warmth from parents and the ability to confide in others about traumatic experiences have been associated with decreased odds of developing IBS, pointing to the potential buffering role of social support and secure attachment (Ju et al., 2020; Low et al., 2020).

Sex differences are a prominent and consistent feature in the epidemiology of IBS and its link to early adversity. IBS is a female-predominant disorder, and women with IBS consistently report higher levels of psychological distress and a greater burden of childhood trauma compared to men (Alsubaie et al., 2022; Marano et al., 2025; Bradford et al., 2012). Meta-analytic data indicate that the association between ACEs and IBS is significant in females but not in males, although some large clinical studies find the association present in both sexes, with differences in the types of trauma most predictive (Joshee et al., 2022; Lee et al., 2025). For women, emotional abuse and household mental illness are frequently identified as significant risk factors, whereas for men, sexual abuse may confer a particularly high risk (Bradford et al., 2012; Lee et al., 2025). The interplay between sex and early trauma extends to neurobiology; neuroimaging studies reveal that while both men and women with IBS and a history of ACEs show alterations in brain networks involved in salience and executive control, men may demonstrate additional ACE-related changes in other neural circuits, such as the cerebellar network (Binod et al., 2025; Gupta et al., 2014). This suggests partially distinct neurobiological pathways linking early stress to adult visceral pain perception across sexes.

Psychological mechanisms are central to understanding how early adversity translates into persistent gastrointestinal symptoms. Clinical studies consistently identify anxiety, depression, alexithymia (difficulty identifying and describing feelings), and somatization as mediators in the pathway from ACEs to IBS (Lee et al., 2025; Rzeszutek et al., 2024; Schubach et al., 2024; Phillips et al., 2013). For example, somatization—the tendency to experience and communicate psychological distress as physical symptoms—has been shown to mediate the relationship between childhood abuse and the severity of abdominal pain and bloating in IBS patients (Schubach et al., 2024). Similarly, anxiety mediates a substantial portion (over 50%) of the effect of ACEs on IBS risk, and resilience factors mediate a smaller but significant portion, particularly in women (Lee et al., 2025). The concept of Disturbance of Self-Organization (DSO), encompassing affective dysregulation and negative self-concept resulting from complex trauma, has also been implicated. DSO symptoms mediate the impact of childhood adversity on IBS symptoms and are associated with greater IBS severity than post-traumatic stress disorder symptoms alone (Sakuma et al., 2024). These findings underscore that the psychological sequelae of early trauma, including maladaptive emotional processing and heightened vigilance towards bodily sensations, are key drivers of symptom chronicity and severity in IBS (Rzeszutek et al., 2024; Jagielski and Riehl, 2021).

The association between ELS and IBS is not limited to psychological trauma but includes a broader range of developmental insults. Perinatal and early childhood health events are recognized risk factors. Lower birth weight has been identified as a significant predictor of IBS in adulthood, potentially reflecting *in utero* programming or being a marker for subsequent developmental challenges (Low et al., 2020; Raslau et al., 2016). Mode of delivery may also influence risk, with some population studies indicating that cesarean section or instrumental vaginal delivery is associated with slightly increased odds of IBS in young adulthood (Olén et al., 2018). Early life medical events, particularly gastrointestinal and other infections, are well-established triggers. A history of gastroenteritis is a known risk factor for post-infectious IBS, and other inflammatory events in childhood, such as infantile urinary tract infections or cow’s milk protein allergy, are associated with a higher prevalence of IBS and other functional gastrointestinal disorders years later (Chogle et al., 2014; Bonilla and Saps, 2013; Tan et al., 2018). These events may act as physiological stressors that, alone or in combination with psychological stress, disrupt the developing brain-gut-microbiota axis and lower the threshold for visceral pain (Zhou et al., 2023; Barreau et al., 2007).

The clinical implications of this evidence are profound. A history of ACEs is associated not only with the presence of IBS but also with a more severe symptom profile, higher levels of comorbid somatic symptoms, poorer quality of life, and greater healthcare utilization (Alsubaie et al., 2022; Priego-Parra et al., 2024; Biggs et al., 2003). Importantly, early adversity is linked to altered physiological stress responses in IBS patients. Individuals with IBS and a history of ACEs demonstrate HPA axis hyperresponsiveness to visceral stressors, such as endoscopy, characterized by higher and more prolonged cortisol release (Videlock et al., 2009). This dysregulated neuroendocrine response is thought to be a key mechanism through which ELS embeds a lasting vulnerability to symptom exacerbation by psychological and visceral stressors. Furthermore, the presence of ACEs complicates the clinical picture, as these patients often exhibit greater psychological distress and may respond differently to therapeutic interventions, although evidence on treatment response modulation is still evolving (Han et al., 2009). Consequently, screening for a history of early adversity and assessing associated psychological factors, such as anxiety and somatization, are increasingly viewed as essential components of a comprehensive, person-centered assessment in IBS, paving the way for integrated treatment approaches that address these foundational risk factors (Woods et al., 2026; Ju et al., 2020; Gordon, 2021).

## 3 Preclinical animal models: recapitulating early life stress-induced visceral hypersensitivity and mechanistic exploration

The robust epidemiological link between ELS and DGBIs, particularly IBS, necessitates experimental models to dissect underlying causal mechanisms. Preclinical animal models of early life adversity have been instrumental in establishing direct causality and providing a controlled platform to unravel the complex, multi-system pathophysiology that links adverse early experiences to lasting visceral pain sensitivity (Accarie and Vanuytsel, 2020; Pohl et al., 2015). These models successfully recapitulate core IBS-like phenotypes, most notably VH, and allow for longitudinal investigation of the neurobiological, endocrine, immune, and microbial sequelae across the lifespan (Prusator et al., 2016; Barreau et al., 2007). The development and characterization of these models represent a critical bridge from clinical correlation to mechanistic understanding.

The most extensively utilized paradigm is the maternal separation (MS) model, where rodent pups are separated from the dam for prolonged periods during a critical postnatal window (O’Mahony et al., 2011; O’Mahony et al., 2009). In preclinical models, this ELS reliably produces a phenotype in adult offspring that mirrors multiple features of IBS, including VH to colorectal distension (CRD), altered colonic motility, increased intestinal permeability, anxiety-like behaviors, and dysregulated stress responses (Tao et al., 2023; Riba et al., 2018; Coutinho et al., 2002). The MS model is considered a robust experimental representation of brain-gut axis dysfunction (O’Mahony et al., 2011). Variations, such as combining unpredictable maternal stress with separation or using the limited bedding/nesting paradigm to induce stress in both dam and pups, also effectively induce lasting visceral hyperalgesia in adult rats and mice (Guo et al., 2015; Moloney et al., 2012; Holschneider et al., 2016). Importantly, these effects are not limited to adulthood; studies demonstrate that MS can induce VH as early as the post-weaning period in mice, indicating that the pathological trajectory begins early in life (Tao et al., 2023; Tao et al., 2021). The translational relevance of these rodent models is supported by analogous findings in larger species. A porcine model of early weaning stress, which mimics psychosocial stress associated with premature separation, similarly results in long-term alterations in ENS function and secretomotor responses, underscoring the cross-species validity of the ELS paradigm (Ziegler and Blikslager, 2017; Medland et al., 2016).

These models have revealed that the predisposition to visceral pain is influenced by genetic background and can be exacerbated by subsequent “hits” in adulthood. For instance, the Lewis rat strain, which exhibits inherent stress sensitivity and susceptibility to inflammation, shows baseline VH and morphological gut alterations compared to other strains, modeling the genetic vulnerability component of IBS (O’Malley et al., 2014). Furthermore, the “two-hit” hypothesis is strongly supported by preclinical data. Animals exposed to ELS display exaggerated VH when challenged with an acute or chronic adult stressor, such as water avoidance stress, a phenomenon particularly pronounced in females (Prusator and Greenwood-Van, 2016a; Winston et al., 2014; Chen et al., 2021a). This synergy is also observed when ELS is combined with transient colonic inflammation, leading to synergistic effects on stress responses and pain behavior (Hasegawa et al., 2023; Hasegawa et al., 2025). Such models effectively capture the clinical reality of symptom exacerbation by later-life stressors in susceptible individuals.

The primary utility of these animal models lies in their capacity for mechanistic dissection. A central finding across studies is the critical involvement of corticotropin-releasing factor (CRF) signaling. ELS alters CRF and CRF receptor type 1 (CRF1R) expression within key brain regions of the emotional-arousal network, including the central amygdala (CeA), bed nucleus of the stria terminalis (avBNST), and paraventricular nucleus (PVN) of the hypothalamus (Huang et al., 2023; Prusator and Greenwood-Van, 2017; Bravo et al., 2011). In females, unpredictable ELS induces VH associated with upregulated CRF and glucocorticoid receptor (GR) in the CeA; knockdown of CeA CRF or CRF1R antagonism reverses this hypersensitivity (Prusator and Greenwood-Van, 2017). Within the PVN, ELS-induced activation of CRF neurons is mediated by complex circuit regulation from the avBNST and by local interactions with activated mast cells that release histamine and other mediators (Huang et al., 2023; Chen Z. et al., 2021; Chen et al., 2023). Systemic or central CRF1R antagonism can attenuate stress-induced VH, confirming the pathway’s therapeutic relevance (Hasegawa et al., 2025).

Neuro-immune interactions, both centrally and peripherally, constitute another major mechanistic axis. In the PVN, mast cell activation and subsequent histamine release contribute to CRF neuronal sensitization and visceral pain (Chen Z. et al., 2021; Chen et al., 2023). In the spinal cord, ELS leads to reduced expression of the glial glutamate transporter EAAT-1, impairing glutamate reuptake and promoting central sensitization; this can be normalized by the drug riluzole (Gosselin et al., 2010). Spinal mechanisms also involve stress-induced epigenetic upregulation of brain-derived neurotrophic factor (BDNF), which is enhanced by estrogen and serotonin signaling in females, contributing to sex-specific pain amplification (Winston et al., 2014; Chen et al., 2021c). Furthermore, downregulation of small-conductance calcium-activated potassium channels (SK2) in the spinal dorsal horn predisposes to VH, which can be rescued by SK2 activation (Wu et al., 2020).

In the peripheral gut, ELS triggers a cascade of events that disrupt the intestinal microenvironment. A key discovery is the expansion of intestinal stem cells and their biased differentiation towards enterochromaffin (EC) cells, driven by nerve growth factor (NGF)-mediated activation of tropomyosin receptor kinase A (TrkA) receptors and crosstalk with Wnt/β-catenin signaling. This leads to increased mucosal serotonin availability, which contributes to visceral hyperalgesia and motility changes (Wong et al., 2019; Chow et al., 2019). The ENS and its glia undergo significant, sexually dimorphic reprogramming. ELS induces a phenotypic switch in enteric glia, particularly in males, making them more resemble the female phenotype, an effect linked to altered prostaglandin and endocannabinoid signaling (Gonzales et al., 2024). ELS also heightens cholinergic secretomotor neuron responses and increases sympathetic nervous system drive, the latter promoting eosinophil infiltration into the colonic mucosa via eotaxin-1 release, thereby contributing to VH (Medland et al., 2016; Duan et al., 2026). Structural changes in enteric glial cells, including in the stomach, are associated with stress-induced motor dysfunction (Tominaga et al., 2016; McClain et al., 2020).

The gut microbiota is a pivotal intermediary in the brain-gut axis dysfunction induced by ELS. MS consistently alters microbial community composition and reduces diversity, changes that persist into adulthood (O’Mahony et al., 2009; Tao et al., 2023; Enqi et al., 2020). These microbial disturbances are functionally significant, as interventions with probiotics or their soluble mediators, prebiotics, or nutritional components like milk fat globule membrane (MFGM) can ameliorate VH and associated behavioral and neuroendocrine abnormalities (Rincel and Darnaudéry, 2020; Collins et al., 2022; O’Mahony et al., 2020; McVey et al., 2020). Interestingly, a maternal high-fat diet during gestation and lactation can paradoxically buffer against the detrimental effects of ELS on visceral pain and anxiety, highlighting complex diet-stress interactions (Rincel et al., 2016).

A paramount insight from these models is the fundamental role of biological sex and ovarian hormones. ELS-induced VH is typically more pronounced or exclusively evident in females, an effect dependent on the activational effects of estradiol (Prusator and Greenwood-Van, 2016a; Chaloner and Greenwood-Van, 2013b; Accarie et al., 2022). Ovariectomy reverses stress-induced visceral pain in females, while estradiol replacement or administration to stressed males reinstates or induces hypersensitivity, respectively (Chen et al., 2021a; Chaloner and Greenwood-Van, 2013b; Accarie et al., 2022). The mechanisms underlying this sex difference involve estrogen-mediated facilitation of spinal BDNF expression, serotonin signaling, and primary afferent neuron sensitization (Chen et al., 2021a; Chen et al., 2021c).

Finally, these preclinical models serve as essential platforms for therapeutic discovery and validation. They have been used to demonstrate the efficacy of diverse agents, including the neuroprotective agent riluzole, the guanylate cyclase-C agonist linaclotide, β3-adrenoceptor agonists, rearranged during transfection kinase inhibitors, natural compounds like *Schisandra chinensis* and gallic acid, and histamine receptor antagonists (Gosselin et al., 2010; Collins et al., 2024; Russell et al., 2019; Ligon et al., 2018; Yang et al., 2012; Guo et al., 2025). These studies not only identify potential novel treatments but also validate specific molecular targets, such as spinal glutamate transporters, enteric β3-adrenoceptors, and CRF1 receptors, within the pathophysiology of ELS-induced visceral pain (Prusator and Greenwood-Van, 2017; Gosselin et al., 2010; Collins et al., 2024). Preclinical models of ELS-induced VH are summarized in Table 1.

**TABLE 1:** Preclinical models of early life stress-induced visceral hypersensitivity.

Model type	Specific paradigm	Species	Stress window	Adult phenotype	Sex differences	Key mechanisms	Clinical relevance	References
Maternal Separation (MS)	Daily separation for 3–6 h, Postnatal Day (PND) 2–14	Rat/Mouse	PND 2-14	• VH • Anxiety-like behavior • Increased intestinal permeability • Enhanced HPA axis reactivity • Gut dysbiosis	Both sexes affected (female phenotype more pronounced)	• CRF system upregulation (PVN/CeA) • EC cell hyperplasia, increased 5-HT • Mast cell activation • Tight junction disruption	Early caregiver neglect	O’Mahony et al. (2009), Moloney et al. (2012), Duan et al. (2026), McClain et al. (2020), Zhang et al. (2016), Tang et al. (2017), Fukui et al. (2018), Sugiyama and Shiotani (2021)
Limited Bedding/Nesting (LBN)	Resource-scarce cage environment, PND 2-9	Rat	PND 2-9	• VH • Somatic hyperalgesia • Anxiety-like behavior	Male-specific	• Fragmented maternal behavior • HPA axis reprogramming • Spinal sensitization	Neglect/Poverty environment	Holschneider et al. (2016), Prusator and Greenwood-Van (2015)
Unpredictable Stress (UNP)	Variable stressors, PND 2-14	Rat	PND 2-14	• VH • Anxiety-like behavior • Depression-like behavior	Female-specific	• Epigenetic changes in amygdala GR/CRF • H3K9 acetylation imbalance • CRF1R-mediated	Unpredictable trauma	Prusator and Greenwood-Van (2016a), Prusator and Greenwood-Van (2017), Chaloner and Greenwood-Van (2013b)
Two-Hit Model	ELS + Adult stress/inflammation	Rat/Mouse	Variable (ELS phase)	• Exacerbated VH • Comorbid anxiety/depression • Gut barrier disruption	Female amplification (two-hit synergy)	• Spinal BDNF epigenetic upregulation • ERα-5-HT3A interaction • CRF1R-mediated	Childhood trauma + adult trigger	Winston et al. (2014), Hasegawa et al. (2025), Chen et al. (2021c), Louwies et al. (2023)
Early Weaning Stress (EWS)	Premature weaning (PND 14-21)	Pig	PND 14-21	• Chronic diarrhea • Gut barrier defects • Motor dysfunction	Female (secretomotor response)	• Cholinergic ENS upregulation • Mast cell activation • Enteric neuroplasticity	Premature maternal separation	Ziegler and Blikslager (2017), Medland et al. (2016), Pohl et al. (2017)
Prenatal Stress (PS)	Restraint/light stress during gestation	Rat	Gestation	• VH • HPA axis alterations • Anxiety-like behavior	Female (BDNF pathway)	• Spinal BDNF epigenetic upregulation • Estrogen-dependent • 5-HT3A-ERα interaction	Maternal stress during pregnancy	Winston et al. (2014), Chen et al. (2021c), Petitfils et al. (2023)
Neonatal Colorectal Distension (CRD)	Mechanical distension of colon, neonatal period	Rat	Neonatal period (e.g., PND 8-21)	• VH (upon adult re-exposure) • Anxiety-like behavior	Documented in both sexes (specific studies vary)	• Hippocampal microglia priming and cytokine release • PVN CRF neuron hyperexcitability • BNST→PVN GABAergic disinhibition	Early-life noxious visceral experience	Zhang et al. (2016), Song et al. (2020), Mahurkar-Joshi and Chang (2020)
Odor-Attachment Learning (OAL)	Classical conditioning: Odor paired with unpredictable shock, PND 8-12	Rat	PND 8-12	• VH	Female-specific (with unpredictable shock)	• Altered neuronal activity in amygdala• Epigenetic reprogramming (GR/CRF)	Attachment to an abusive caregiver	Prusator and Greenwood-Van (2017), Louwies et al. (2023), Louwies and Greenwood-Van (2020)
Neonatal Colon Inflammation (NCI)	Chemical irritant (e.g., TNBS) induced colitis, neonatal period	Mouse	Neonatal period	• VH in adolescence/adulthood	Studies in both sexes	• Suppression of auditory cortex (Au1) GABAergic neurons • Cross-modal neural circuit reorganization	Early-life inflammatory insult	Yu et al. (2026)

Abbreviations: 5-HT, serotonin; Au1, Primary auditory cortex; BDNF, Brain-derived neurotrophic factor; BNST, bed nucleus of the stria terminalis; CeA, central nucleus of the amygdala; CRD, colorectal distension; CRF, Corticotropin-releasing factor; CRF1R, Corticotropin-releasing factor receptor type 1; EC, enterochromaffin cell; ELS, early life stress; ENS, enteric nervous system; ERα, estrogen receptor alpha; GABA, Gamma-aminobutyric acid; GR, glucocorticoid receptor; H3K9, Histone three lysine 9; HPA, Hypothalamic-pituitary-adrenal; IBS, irritable bowel syndrome; MS, maternal separation; NCI, neonatal colon inflammation; OAL, Odor-attachment learning; PND, postnatal day; PVN, paraventricular nucleus of the hypothalamus; TNBS, 2,4,6-Trinitrobenzenesulfonic acid; VH, visceral hypersensitivity.

## 4 Neuro-endocrine-immune pathways: from stress perception to central pain amplification

The therapeutic targets identified in preclinical models emerge from a profound dysregulation of interconnected neuro-endocrine-immune pathways, which form the core biological substrate translating early life adversity into persistent VH (Buckley et al., 2014; O’Malley et al., 2011). This pathophysiological axis represents a maladaptive cascade, initiated by the perception of stress and culminating in the central amplification of nociceptive signals from the gut. The dysregulated stress response, frequently observed in individuals with a history of ELS, serves as the principal orchestrator of this process (Greenwood-Van and Johnson, 2017; O’Mahony et al., 2011). The HPA axis is a primary mediator, where ELS can induce long-lasting alterations in its set point and reactivity (Fuentes and Christianson, 2018; Videlock et al., 2009). Studies in IBS patients demonstrate that a history of early adverse life events is associated with HPA axis hyperresponsiveness to visceral stressors, an effect more closely linked to the trauma history than to the IBS diagnosis itself (Videlock et al., 2009). This programming effect is evident even prenatally, where maternal stress can influence fetal HPA axis development, as reflected in newborn hair cortisol levels (Romero-Gonzalez et al., 2018). The central nucleus (CeA) of the amygdala is a critical hub where this dysregulated neuroendocrine signaling converges to modulate visceral pain perception. In adult females with a history of unpredictable ELS, VH is associated with increased expression of both GR and CRF mRNA within the CeA (Prusator and Greenwood-Van, 2017). Antagonism of CRF1R in the CeA can attenuate this hypersensitivity, highlighting the pivotal role of amygdalar CRF signaling (Prusator and Greenwood-Van, 2017). The enduring nature of these changes is thought to be underpinned by epigenetic mechanisms, where stress-induced histone acetylation at the promoter regions of GR and CRF genes in the CeA contributes to visceral pain susceptibility (Louwies et al., 2023; Guan et al., 2022). In a two-hit model of ELS followed by adult stress, predictable ELS-conferred resilience is lost after adult stress, an event associated with reduced H3K9 acetylation at the GR promoter and increased acetylation at the CRF promoter in the CeA of female rats; these epigenetic alterations and the resulting exacerbation of VH can be modulated by histone deacetylase (HDAC) inhibition (Louwies et al., 2023).

Beyond the amygdala, ELS induces widespread functional and structural reorganization across a central pain amplification network. Neuroimaging studies in humans and autoradiography in rodent models reveal that ELS alters brain activation patterns during visceral stimulation, enhancing responses in regions like the insula, anterior cingulate cortex (ACC), amygdala, and hippocampus (Holschneider et al., 2016; Wouters et al., 2012). In female IBS patients, gray matter volume reductions in areas including the amygdala, insula, and ACC are correlated with early trauma scores (Labus et al., 2014). Furthermore, ELS shapes intrinsic brain connectivity, with alterations observed in resting-state networks such as the salience/executive control network, which is implicated in central pain amplification (Gupta et al., 2014). These central alterations exhibit sexual dimorphism, with ELS-related changes in functional connectivity patterns differing between male and female patients with chronic abdominal pain (Gupta et al., 2014). The hippocampus, a region integral to context processing and stress modulation, is also profoundly affected. In rodent models, ELS increases the expression of circular RNA circKcnk9 in the hippocampal CA1 region, which acts as a sponge for miR-124-3p, leading to increased expression of EZH2 and enhanced hippocampal long-term potentiation, thereby contributing to VH and comorbid anxiety (Liu et al., 2022). Functional segmentation of the hippocampus reveals distinct roles, with dorsal hippocampal circuits modulating both visceral sensitivity and anxiety, while ventral hippocampal circuits primarily influence anxiety behaviors (Lin et al., 2024). ELS can also sensitize hippocampal microglia, such that a later adult challenge precipitates cytokine release, downregulates hippocampal GR expression, and fosters VH (Zhang et al., 2016).

The transmission and amplification of nociceptive signals at the spinal cord level constitute another critical node. In rodent models, ELS has been shown to lead to epigenetic upregulation of BDNF in the lumbosacral spinal cord, a mechanism more prominent in female offspring and exacerbated by a second stress in adulthood (Winston et al., 2014). This BDNF upregulation is driven by a serotonin-estrogen interaction in females, where stress-induced increases in cerebrospinal fluid serotonin activate spinal 5-HT3A receptors, leading to increased spinal estrogen receptor alpha (ERα) expression and subsequent pro-transcriptional epigenetic modifications at the BDNF promoter (Chen et al., 2021c). Furthermore, ELS impairs spinal glutamate reuptake mechanisms, evidenced by reduced expression of the astrocytic excitatory amino acid transporter EAAT1 (GLT-1); pharmacological activation of glutamate transport with riluzole normalizes ELS-induced VH (Gosselin et al., 2010). Spinal cord sensitization is also reflected in enhanced phosphorylation of extracellular signal-regulated kinase (ERK) 1/2 in the dorsal horn in response to colorectal distension following stress (Noor-Mohammadi et al., 2021).

The neuro-endocrine cascade exerts powerful effects on peripheral effectors via autonomic and neuroimmune pathways, which in turn feed back to sustain central sensitization. ELS is associated with altered autonomic function, particularly sympathetic overactivation (Duan et al., 2026; Tillisch et al., 2005). This sympathetic overdrive has been shown in animal models to contribute to pathology by driving eosinophil infiltration into the colonic mucosa through noradrenergic signaling and the release of eotaxin-1 from mesenchymal cells (Duan et al., 2026). Pharmacological or chemogenetic inhibition of this sympathetic overactivation attenuates both mucosal immune changes and VH (Duan et al., 2026). Mast cells are key cellular mediators at the neuro-immune interface. ELS increases mast cell numbers and proximity to enteric ganglia and neurons (McClain et al., 2020). Activated mast cells release mediators like histamine and NGF, which can sensitize adjacent primary afferents and enteric neurons (Chen Z. et al., 2021; Chen et al., 2023; Tsang et al., 2012). In the PVN of the hypothalamus, mast cell-derived histamine activates CRF neurons via histamine H2 receptors, contributing to stress-induced VH (Chen et al., 2023). This mast cell-neuron interaction is bidirectional, as NGF released under stress conditions not only activates mast cells but also induces neuronal plasticity in dorsal root ganglia and the spinal cord, lowering pain thresholds (Tsang et al., 2012; Barreau et al., 2004). ELS also alters the functional interactions between mast cells and enteric glia, potentially via histamine signaling, suggesting a role for enteric neuroplasticity in long-term gut dysfunction (McClain et al., 2020).

The integration of these pathways often occurs within discrete neural circuits that gate pain and stress responses. A critical circuit involves the bed nucleus of the stria terminalis (BNST) and the PVN. In a MS model, susceptible mice exhibit activation of glutamatergic projections or inhibition of GABAergic projections from the anteroventral BNST (avBNST) to PVN CRF neurons, whereas resilient mice show adaptive restoration of SK2 channel function in these neurons (Huang et al., 2023). Conversely, disinhibition of PVN-projecting GABAergic neurons in the avBNST, leading to increased excitability of PVN CRF neurons, has been identified as a mechanism underlying VH in another ELS model (Song et al., 2020). This highlights the complexity and potential duality of BNST-PVN circuitry in determining vulnerability or resilience. Peripheral CRF signaling is another integrative mechanism. Stress increases the expression and activation of colonic CRF1 and CRF2 receptors, and ELS alters how these receptors respond to subsequent acute psychological or physical stressors (O’Malley et al., 2010). Systemic administration of a CRF1R antagonist can attenuate VH induced by the combination of ELS and prior colitis, confirming the therapeutic relevance of this pathway (Hasegawa et al., 2025).

**FIGURE 1:**
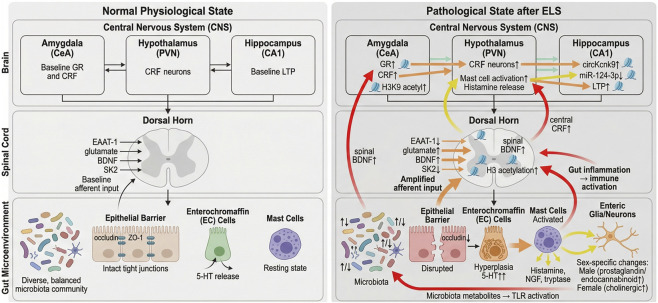
Integrated mechanisms of early life stress-induced visceral hypersensitivity along the brain-gut axis. This schematic illustrates the multi-level pathophysiology by which early life stress (ELS) programs lasting visceral hypersensitivity (VH). The figure is organized from top to bottom along the brain-gut axis, with left panels representing normal physiological states and right panels depicting ELS-induced pathological alterations. Central nervous system (CNS): ELS induces epigenetic modifications in the central amygdala (CeA), characterized by increased histone H3K9 acetylation at glucocorticoid receptor (GR) and corticotropin-releasing factor (CRF) promoters, leading to GR/CRF upregulation. In the paraventricular nucleus (PVN) of the hypothalamus, ELS activates resident mast cells, which release histamine to stimulate CRF neurons via H2 receptors. In the hippocampal CA1 region, ELS upregulates circular RNA circKcnk9, which sponges miR-124-3p, leading to increased EZH2 expression and enhanced long-term potentiation (LTP). Spinal cord: ELS downregulates the astrocytic glutamate transporter EAAT-1, impairing glutamate reuptake and promoting central sensitization. Concurrently, ELS induces epigenetic upregulation of brain-derived neurotrophic factor (BDNF) via histone H3 acetylation, a mechanism amplified by estrogen-serotonin signaling in females. Downregulation of small-conductance calcium-activated potassium channels (SK2) further increases dorsal horn neuron excitability. Gut microenvironment: ELS disrupts intestinal homeostasis through multiple mechanisms: (1) Microbiota: ELS induces gut dysbiosis characterized by reduced diversity and altered metabolite profiles. (2) Epithelial barrier: Tight junction proteins (occludin, ZO-1) are downregulated, increasing intestinal permeability. (3) Stem cell niche: Nerve growth factor (NGF) activates TrkA receptors on intestinal stem cells, transactivating Wnt/β-catenin signaling to drive differentiation toward enterochromaffin (EC) cells. (4) EC cells: EC cell hyperplasia leads to increased serotonin (5-HT) bioavailability, sensitizing afferent nerve terminals. (5) Mast cells: ELS increases mast cell number and activation, releasing histamine, NGF, and tryptase. (6) Enteric glia: ELS induces a sex-dependent phenotypic switch in enteric glia (male-specific), mediated by prostaglandin and endocannabinoid signaling. (7) Enteric neurons: Cholinergic secretomotor neuron responses are upregulated (female-specific), and sympathetic overactivation drives eosinophil infiltration via eotaxin-1. Vicious cycle: Peripheral sensitization feeds back to amplify central pain processing. Gut-derived 5-HT, immune mediators, and microbial products activate afferent pathways, reinforcing spinal BDNF upregulation and amygdala CRF signaling, thereby perpetuating visceral hypersensitivity. Red bold arrows indicate the self-sustaining maladaptive cycle. CRF, corticotropin-releasing factor; GR, glucocorticoid receptor; PVN, paraventricular nucleus; CeA, central amygdala; LTP, long-term potentiation; EAAT, excitatory amino acid transporter; BDNF, brain-derived neurotrophic factor; SK2, small-conductance calcium-activated potassium channel type 2; EC, enterochromaffin; 5-HT, serotonin; NGF, nerve growth factor; TrkA, tropomyosin receptor kinase A; ENS, enteric nervous system; TLR, Toll-like receptor; VH: visceral hypersensitivity. Created with BioRender.com.

Ultimately, the convergence of these neuro-endocrine-immune disturbances creates a state of heightened alert within the brain-gut axis, where visceral signals are disproportionately amplified. The sensitization of primary afferent pathways is mediated by factors such as calcitonin gene-related peptide (CGRP) and growth factor receptors like RET (Russell et al., 2019; Noor-Mohammadi et al., 2021). Inhibition of peripheral CGRP signaling with a monoclonal antibody reverses stress-induced colonic hypersensitivity and associated spinal ERK phosphorylation, implicating CGRP in central sensitization (Noor-Mohammadi et al., 2021). Similarly, inhibition of the RET kinase attenuates VH across multiple models, highlighting a role for glial cell line-derived neurotrophic factor family signaling in nociception (Russell et al., 2019). This intricate, multi-level dysregulation, spanning from epigenetic modifications in limbic circuits to neuroimmune activation in the gut wall, defines the biological signature of ELS-induced visceral pain and provides a roadmap for targeted therapeutic interventions. To provide conceptual clarity regarding the relationship among these diverse mechanisms, I propose that they form a hierarchical, self-reinforcing network. At the apex, ELS triggers dysregulation of the HPA axis and CRF signaling within limbic circuits, which act as primary drivers. At the intermediate level, these central alterations engage secondary amplifiers. These include spinal sensitization (BDNF upregulation, EAAT-1 dysfunction, SK2 downregulation), peripheral immune activation (mast cells, eosinophils), EC hyperplasia with serotonin overproduction, and gut dysbiosis. At the effector level, these changes culminate in VH, epithelial barrier dysfunction, and pain-related behaviors, which in turn feed back to perpetuate central dysregulation, thereby creating a self-sustaining cycle (Figure 1).

## 5 Peripheral effectors and the gut microenvironment: epithelial barrier, enteric nervous system, immune cells, and microbiota

The central amplification of visceral signals is critically dependent on and driven by profound alterations at the peripheral interface—the gut itself. The colonic mucosa, its intricate nervous system, resident immune cells, and the luminal microbial community constitute a dynamic microenvironment that is fundamentally reshaped by ELS. These changes collectively lower the threshold for nociceptive signaling, creating a persistent state of peripheral sensitization that feeds into and sustains the central dysregulation.

A pivotal cell type linking the luminal environment to neural signaling is the EC cell. ELS, particularly in the form of neonatal MS, induces a lasting expansion of the intestinal stem cell compartment, driving increased differentiation toward secretory lineages, including EC cells (Wong et al., 2019). This results in EC hyperplasia and a significant increase in the synthesis and bioavailability of peripheral serotonin (5-hydroxytryptamine, 5-HT) (Wong et al., 2019; Tao et al., 2022; Yang et al., 2024). The surplus of intestinal 5-HT is a key driver of VH and motility disturbances (Tao et al., 2022; Yang et al., 2024). Mechanistically, this EC-5-HT axis is activated by NGF signaling via the TrkA. NGF, whose expression is elevated by ELS, transactivates the Wnt/β-catenin pathway in intestinal stem cells, promoting their proliferation and differentiation into EC cells (Wong et al., 2019; Chow et al., 2019). This pathway establishes a direct link from psychological stress to a physical alteration in gut epithelial composition. The increased 5-HT not only affects visceral sensation but also, through activation of the 5-HT4 receptor, activates the Wnt pathway within the ENS, promoting neurogenesis and altering the density of the intestinal neural network, thereby further dysregulating gut function (Yang et al., 2024).

Concurrently, the ENS itself undergoes significant stress-induced neuroplasticity. The enteric glial cells (EGCs), essential for ENS support and function, exhibit structural and phenotypic changes. In models of combined early and adult stress, gastric EGCs show increased process area overlapping with neurons and morphological alterations, which correlate with delayed gastric emptying (Tominaga et al., 2016). A seminal finding reveals a sex-dependent phenotypic switch in colonic EGCs following ELS. Male glia undergo a transformation toward a phenotype typically observed in females, characterized by alterations in genes related to inflammation and cellular communication, which precedes physiological gut dysfunction mirroring DGBIs (Gonzales et al., 2024). These glial changes are modulated by prostaglandin and endocannabinoid signaling (Gonzales et al., 2024). Furthermore, ELS increases the potential for histamine-dependent interactions between mast cells and EGCs. Myenteric glia exhibit calcium responses to mast cell mediators, an effect blocked by histamine H1 receptor antagonists, and ELS alters this glial-mast cell cross-talk, contributing to enteric neuroplasticity (McClain et al., 2020). In a translational porcine model of early weaning stress, ELS induces a lasting, sex-specific upregulation of the cholinergic ENS, with females showing heightened secretomotor neuron responses, providing a mechanistic link to increased susceptibility to gastrointestinal diseases (Medland et al., 2016).

Immune activation, particularly involving mast cells, is a cornerstone of the ELS-induced peripheral pathophysiology. Mast cell numbers and activity are increased in the gut mucosa of stressed animals (Pohl et al., 2017). These cells often lie in close apposition to enteric nerves, and their activation releases a plethora of mediators that can directly sensitize afferent neurons (Philpott et al., 2011). Critically, mast cell activation is not confined to the gut. In the hypothalamic PVN, a key hub for stress integration, neonatal MS triggers the activation of resident mast cells. These PVN mast cells release proinflammatory mediators, including histamine, which activates neighboring CRF neurons via histamine H2 receptors, ultimately driving VH (Chen Z. et al., 2021; Chen et al., 2023). This pathway can be inhibited by mast cell stabilizers or H2 receptor antagonists infused directly into the PVN (Chen Z. et al., 2021; Chen et al., 2023). Additionally, sympathetic nervous system overactivation, a consequence of ELS, drives eosinophil infiltration into the colonic mucosa via noradrenergic signaling and the release of eotaxin-1 from mesenchymal cells, another immune pathway contributing to visceral pain (Duan et al., 2026).

Integrity of the intestinal epithelial barrier is consistently compromised by ELS, leading to increased intestinal permeability. This defect is observable from weaning into adulthood (Tao et al., 2023; Riba et al., 2018; Pohl et al., 2017; Moussaoui et al., 2017). The barrier dysfunction is mediated, in part, by NGF, which can alter mucosal integrity (Barreau et al., 2004). Stress-induced permeability allows for increased translocation of microbial components and dietary antigens, which can engage pattern recognition receptors on immune and epithelial cells. Indeed, ELS upregulates the expression of Toll-like receptors (TLRs), key sentinels of the innate immune system, in the colonic mucosa (McKernan et al., 2009). Specifically, TLR5 expression is increased in colonocytes of MS mice that develop colonic hypersensitivity, and this is associated with a dysbiotic expansion of flagellated bacteria; acute administration of the TLR5 agonist flagellin can induce transient VH in naïve mice (Mallaret et al., 2022). TLR4 signaling in the PVN microglia also plays a crucial role in mediating MS-induced VH, linking peripheral immune triggers to central stress circuits (Tang et al., 2017). Pharmacological restoration of the gut barrier during the neonatal stress period, using a myosin light chain kinase inhibitor, can partially reverse long-term behavioral and neuroendocrine consequences of MS, underscoring the critical role of early barrier function in programming adult outcomes (Rincel et al., 2019).

The gut microbiota is both a target and a mediator of ELS effects. Stressed animals display altered microbial composition (dysbiosis) characterized by reduced diversity, changes in the abundance of specific taxa, and an altered functional metabolic profile (Tao et al., 2023; Riba et al., 2018; Enqi et al., 2020; Moussaoui et al., 2017; Petitfils et al., 2023). This dysbiosis is evident early in life and can precede and predict adult behavioral phenotypes, including resilient versus comorbid gut-brain outcomes, in a sex-dependent manner (Wilmes et al., 2024). The microbial alterations contribute to pathophysiology through multiple mechanisms. They can disrupt the production of bioactive metabolites, such as GABA-containing bacterial lipopeptides, which have analgesic properties; a decrease in these metabolites is linked to VH in both prenatally stressed mice and patients with IBS (Petitfils et al., 2023). Dysbiosis also fuels low-grade immune activation and barrier defects. Furthermore, specific microbial signatures in IBS patients correlate with both a history of early life trauma and alterations in brain structure, highlighting the integrative role of the microbiome within the brain-gut axis (Labus et al., 2017).

These peripheral effectors do not operate in isolation but engage in continuous, maladaptive crosstalk. Increased intestinal permeability permits bacterial products to activate mucosal immune cells and TLRs, which release mediators that sensitize the ENS and primary afferent nerve terminals. The sensitized ENS and excess 5-HT from EC cells further dysregulate motility and secretion, altering the luminal microenvironment and exacerbating dysbiosis. This creates a self-perpetuating cycle that maintains peripheral sensitization and feeds aberrant signals to the central nervous system. Interventions targeting these peripheral pathways, such as specific probiotics, prebiotics, dietary supplements like MFGM, or even silicon-rich mineral water activating the glucagon-like peptide (GLP)2-Wnt1 axis for epithelial repair, have shown efficacy in ameliorating ELS-induced VH and associated microbiota shifts, offering promising therapeutic avenues rooted in modulating the gut microenvironment (Rincel and Darnaudéry, 2020; Collins et al., 2022; O’Mahony et al., 2020; McVey et al., 2020; Chen et al., 2024; Fukui et al., 2018).

## 6 Epigenetic mechanisms: the molecular interface between early experience and lasting phenotype

The self-perpetuating cycle of peripheral sensitization in the gut is not merely a transient state but often reflects a stable, maladaptive reprogramming of physiological systems. This enduring change, whereby a transient ELS event instigates a lifelong predisposition to VH, is fundamentally mediated by epigenetic mechanisms. Epigenetics represents a candidate molecular machinery. It translates environmental exposures, such as ELS, into lasting alterations in gene expression without changing the DNA sequence itself. As such, it serves as potential interface between early experience and adult phenotype in DGBIs, a concept derived primarily from preclinical studies (Mahurkar-Joshi and Chang, 2020; Dinan et al., 2010). Although epigenetic mechanisms provide a compelling explanatory framework, their stability, tissue specificity, and translational relevance in humans remain to be fully established. The involvement of DNA methylation, histone modifications, and non-coding RNAs provides a plausible biological substrate for the “memory” of adverse early experiences, influencing stress responsiveness, pain processing, neuroendocrine function, and peripheral gut physiology long after the initial insult has ceased (Louwies et al., 2023; Mahurkar-Joshi and Chang, 2020).

Central epigenetic dysregulation within key nodes of the brain-gut axis is a pivotal mechanism underlying ELS-induced VH. The amygdala, particularly the CeA, is a major site for this programming. Studies using predictable versus unpredictable ELS paradigms in rats reveal that sex-specific vulnerability to visceral pain is linked to histone acetylation states in the CeA. In adult female rats, unpredictable ELS leads to increased histone three lysine 9 (H3K9) acetylation at the promoter of the GR gene and altered GR binding at the CRF promoter, correlating with VH (Louwies et al., 2023; Louwies and Greenwood-Van, 2020). These changes effectively “memorize” the ELS event, dysregulating the central stress response. Pharmacologically modulating histone acetylation in the CeA can reverse this phenotype; infusion of the HDAC inhibitor trichostatin A (TSA) attenuates stress-induced VH (Louwies et al., 2023), while inhibition of histone acetyltransferases (HAT) with garcinol can normalize acetylation and GR binding (Louwies and Greenwood-Van, 2020). Similarly, in the spinal cord, a crucial site for visceral pain transmission, ELS is associated with altered histone acetylation patterns. MS increases histone acetylation in the lumbosacral spinal cord, and systemic administration of the HDAC inhibitor suberoylanilide hydroxamic acid (SAHA) reverses both VH and anxiety-like behaviors (Moloney et al., 2015). Furthermore, chronic prenatal stress epigenetically upregulates BDNF expression in the spinal dorsal horn of female offspring via increased histone H3 acetylation and RNA polymerase II binding at the BDNF promoter, contributing to a sex-specific exacerbation of visceral pain (Winston et al., 2014).

Beyond transcriptional regulation via histone modifications, epigenetic mechanisms also exert precise control over specific ion channels and transporters involved in neuronal excitability. In the spinal dorsal horn, ELS-induced VH is associated with the downregulation of the SK2. This downregulation leads to increased neuronal firing rates, and pharmacologically activating SK2 channels can reverse both the electrophysiological alterations and the behavioral VH (Wu et al., 2020). Additionally, excitatory amino acid transporter (EAAT) function in the anterior cingulate cortex and spinal cord is dysregulated by ELS and fluctuates across the estrous cycle, contributing to central sensitization to visceral pain (Moloney et al., 2016).

The reach of epigenetic programming extends beyond the central nervous system to the peripheral gut environment, influencing the very effectors that sustain hypersensitivity. While direct evidence for epigenetic changes in enteric neurons or epithelial cells following ELS is an emerging area, the long-term alterations in gut barrier function and immune tone are consistent with stable reprogramming. The enduring increase in intestinal permeability induced by neonatal stress can be prevented by pharmacologically inhibiting myosin light chain kinase (MLCK) during the stress period, an intervention that also normalizes adult stress responses and microbiota composition (Rincel et al., 2019). This suggests that early, transient targeting of a peripheral pathway can have lasting benefits, potentially by preventing the initiation of a maladaptive epigenetic cascade. Furthermore, dietary interventions such as MFGM and prebiotics, which ameliorate ELS-induced VH, also induce significant and lasting changes in gut microbiota composition and brain gene expression, indicating they may act, in part, by modifying epigenetic landscapes (O’Mahony et al., 2020).

The establishment and maintenance of these epigenetic marks are highly context-dependent, influenced by factors such as sex, stressor characteristics, and later life experiences, which align with the clinical heterogeneity of IBS. Sex differences are pronounced, with female rodents showing greater susceptibility to epigenetic dysregulation in the CeA and spinal cord following unpredictable ELS or a two-hit stress model (Winston et al., 2014; Louwies et al., 2023; Louwies and Greenwood-Van, 2020). The predictability of the stressor itself is a critical variable; predictable ELS can induce resilience in females, but this resilience is lost following adult chronic stress, accompanied by distinct epigenetic changes in the CeA (Louwies et al., 2023). This two-hit model, involving ELS followed by adult stress, effectively recapitulates the clinical scenario where childhood adversity predisposes individuals to develop functional disorders after a subsequent trigger, with epigenetic dysregulation at its core (Winston et al., 2014; Louwies et al., 2023). Even the prenatal environment contributes to this programming, as maternal stress can impart epigenetic changes in the offspring (Winston et al., 2014; Romero-Gonzalez et al., 2018).

Human studies corroborate the translational significance of these preclinical findings. Multi-omics analyses in women with IBS and a history of ACEs reveal unique biosignatures encompassing brain network connectivity, gut microbiome composition, and clinical symptom severity (Binod et al., 2025). This systems-level dysregulation is consistent with broad developmental reprogramming. Furthermore, experiences of discrimination in adulthood are associated with distinct gut microbiome profiles and mucosal transcriptomic changes, suggesting that social stressors can also engage pathways that may interface with epigenetic regulation of gut physiology (Dong et al., 2024). These human data underscore that epigenetic mechanisms, shaped by a confluence of early and later life events, create a biological embedding of stress that manifests as persistent gut-brain axis dysfunction.

The reversible nature of many epigenetic marks opens promising therapeutic avenues. Pharmacological agents like HDAC inhibitors have demonstrated efficacy in preclinical models (Moloney et al., 2015). Perhaps more translatable are nutritional and microbial interventions. The combination of MFGM and prebiotics not only ameliorates VH but also modulates HPA axis reactivity, suggesting a multi-system impact (O’Mahony et al., 2020). The demonstrable plasticity of the epigenome indicates that the deleterious programming by ELS is not a life sentence. Future research must further delineate the precise epigenetic signatures in specific cell types across the lifespan and in response to interventions. Unraveling this molecular interface will be key to developing mechanistically targeted strategies that can reset the maladaptive programs established by early adversity.

## 7 Sex differences and the concept of resilience: modifiers of risk and trajectory

The preceding discussion on epigenetic plasticity underscores that the pathogenic trajectory from ELS to VH is not uniform. A critical layer of complexity is introduced by biological sex, which fundamentally shapes susceptibility, and by the concept of resilience, which explains why not all individuals exposed to adversity develop chronic pain. Epidemiological data robustly establish IBS as a female-predominant disorder (Marano et al., 2025; Lee et al., 2025), and clinical studies consistently find that women with IBS report higher levels of psychological distress (Marano et al., 2025). This sex disparity is mirrored in preclinical models, where the effects of ELS on visceral pain are often more pronounced or exclusive to females. For instance, unpredictable ELS paradigms reliably induce lasting VH in adult female, but not male, rats (Prusator and Greenwood-Van, 2016a; Chaloner and Greenwood-Van, 2013b; Louwies and Greenwood-Van, 2020). This sexual dimorphism is highly dependent on the nature of the ELS. While unpredictable stress may preferentially affect females, other paradigms like MS or limited nesting can induce visceral and somatic hypersensitivity specifically in adult male rats (Prusator and Greenwood-Van, 2016b; Prusator and Greenwood-Van, 2015). This indicates that sex-related vulnerability or resilience is directly associated with the specific characteristics of the adverse experience (Prusator and Greenwood-Van, 2016b).

The mechanisms underlying this sexual dimorphism are multifaceted, with ovarian hormones, particularly estradiol, playing a pivotal activational role. In female rodents, ovariectomy reverses ELS-induced VH, while estradiol replacement reinstates it (Chaloner and Greenwood-Van, 2013b; Accarie et al., 2022). Estrogen contributes to the exacerbation of visceral pain in “two-hit” stress models, where chronic prenatal stress followed by adult stress leads to a female-specific, aggravated VH (Winston et al., 2014; Chen et al., 2021c). This effect is mediated through estrogen ERα in the spinal cord, which interacts with serotonin signaling to upregulate BDNF (Chen et al., 2021c). Furthermore, estrous cycle fluctuations influence visceral pain sensitivity and central glutamatergic systems, effects that are dysregulated by prior ELS (Moloney et al., 2016). The impact of sex extends beyond central circuits to the periphery. Enteric glia exhibit intrinsic sexual dimorphism, and ELS can induce a phenotypic switch in male glia toward a female-like state, concomitant with changes in gut physiology (Gonzales et al., 2024). Autonomic responses to visceral stressors in IBS patients also show sex-specific alterations, with male patients displaying more pronounced sympathetic predominance (Tillisch et al., 2005).

Despite the heightened risk associated with female sex and ELS, a significant proportion of exposed individuals do not develop chronic visceral pain, highlighting the importance of resilience. In the context of ELS and visceral pain, it manifests as a phenotypic state distinct from comorbid pain and anxiety. A preclinical study using cluster analysis identified four distinct adult phenotypes following MS: resilient, pain-predominant, immobile-predominant, and comorbid (Wilmes et al., 2024). Crucially, the gut microbiota signature in early life already differed in a sex-dependent manner according to these eventual adult phenotypes, suggesting that early microbial and metabolic pathways may contribute to resilient versus susceptible trajectories (Wilmes et al., 2024). Clinically, resilience acts as a protective mediator. In a large cohort, while anxiety mediated a substantial portion of the relationship between ACEs and IBS, resilience also mediated a significant part of this effect (Lee et al., 2025). Other protective factors include benevolent childhood experiences (BCEs), where a higher number of BCEs is associated with reduced odds of developing IBS (Rojas et al., 2025), and the act of confiding in others about traumatic events, which decreases the likelihood of having IBS (Ju et al., 2020).

The neurobiological substrates of resilience are beginning to be elucidated. Patients with IBS show reduced scores on both perceived and relative resilience measures compared to healthy controls (Kilpatrick et al., 2024). Neuroimaging reveals that these lower resilience scores are associated with reduced resting-state connectivity within brain networks crucial for maintaining homeostasis, such as the central autonomic network (Kilpatrick et al., 2024). Furthermore, the detrimental impact of ELS on brain structure is modulated by an interaction between genetic polymorphisms and the history of ELS (Gupta et al., 2016). This gene-environment interaction may partly determine resilient versus susceptible neurophenotypes. Resting-state network alterations linked to ELS also show sex-specific patterns (Gupta et al., 2014).

**FIGURE 2:**
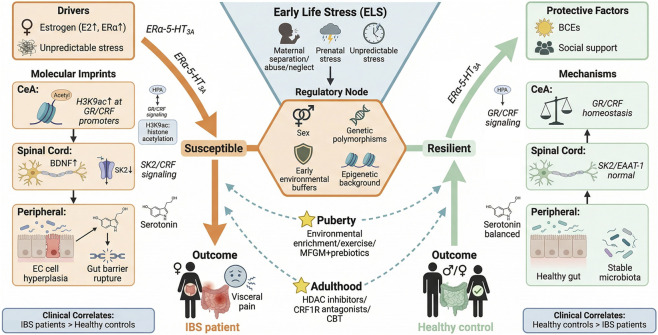
Sex dimorphism and resilience mechanisms determine divergent trajectories following early life stress. This schematic illustrates the Y-shaped framework explaining why individuals exposed to similar early life stress (ELS) develop distinct outcomes: visceral hypersensitivity (VH) (susceptible pathway) versus normal pain perception (resilient pathway). Common starting point: ELS (maternal separation, abuse, neglect, or prenatal stress) serves as the initial insult. Regulatory node: The trajectory diverges at a regulatory node influenced by multiple factors including biological sex, genetic polymorphisms, early environmental buffers (e.g., benevolent childhood experiences, social support), and epigenetic background. Left panel (Susceptible pathway): Predominantly observed in females, driven by activational effects of estrogen (E2) and estrogen receptor alpha (ERα) upregulation. Key molecular imprints include: (1) Central amygdala (CeA): Increased histone H3K9 acetylation at glucocorticoid receptor (GR) and corticotropin-releasing factor (CRF) promoters, leading to GR/CRF expression imbalance and HPA axis hyperreactivity. (2) Spinal cord: Epigenetic upregulation of brain-derived neurotrophic factor (BDNF) via histone H3 acetylation, enhanced by ERα-5-HT3A receptor interaction; downregulation of small-conductance calcium-activated potassium channels (SK2) and excitatory amino acid transporter-1 (EAAT-1). (3) Hippocampus: Upregulation of circular RNA circKcnk9, sponging miR-124-3p, leading to enhanced long-term potentiation (LTP). (4) Peripheral alterations: Enterochromaffin (EC) cell hyperplasia with increased serotonin (5-HT) bioavailability; mast cell activation; gut barrier disruption; dysbiosis; sex-specific enteric glial phenotypic switch (male) and cholinergic enteric nervous system upregulation (female). Phenotypic outcomes include VH, anxiety-like behavior, gut hyperpermeability, and comorbid depression. Right panel (Resilient pathway): Associated with protective factors including high benevolent childhood experiences (BCEs), social support, and predictable stress environments. Key protective mechanisms include: (1) Central: Maintained epigenetic homeostasis at GR/CRF promoters in CeA; normal HPA axis function; preserved spinal SK2 and EAAT-1 expression; normal hippocampal LTP. (2) Peripheral: Normal EC cell number and 5-HT levels; quiescent mast cells; intact gut barrier; stable microbiota; normal enteric glial and neuronal function. Phenotypic outcomes include normal pain threshold, normal emotional behavior, and intact gut function. Clinical correlates: Susceptible pathway corresponds to IBS patients (particularly females, high ACEs scores, high psychological comorbidity); resilient pathway corresponds to ELS-exposed healthy controls (high BCEs scores, high psychological resilience). Intervention windows: Key developmental windows (prepuberty/puberty) and adulthood offer opportunities to redirect susceptible trajectories toward resilient outcomes through environmental enrichment, exercise, nutritional interventions (MFGM + prebiotics), HDAC inhibitors, CRF1R antagonists, and psychotherapies (CBT, music therapy). Dashed arrows indicate the redirecting effect of interventions. ACEs, adverse childhood experiences; BCEs, benevolent childhood experiences; E2, estradiol; ERα, estrogen receptor alpha; GR, glucocorticoid receptor; CRF, corticotropin-releasing factor; HPA, hypothalamic-pituitary-adrenal; BDNF, brain-derived neurotrophic factor; 5-HT3A, serotonin receptor 3A; SK2, small-conductance calcium-activated potassium channel type 2; EAAT-1, excitatory amino acid transporter-1; LTP, long-term potentiation; EC, enterochromaffin; 5-HT, serotonin; ENS, enteric nervous system; MFGM, milk fat globule membrane; HDAC, histone deacetylase; CRF1R, corticotropin-releasing factor receptor 1; CBT, cognitive behavioral therapy; VH: visceral hypersensitivity. Created with BioRender.com.

The plasticity implied by the concept of resilience opens avenues for intervention, even after ELS exposure. Enriched environmental housing during critical developmental periods like prepuberty and puberty can effectively prevent or reverse ELS-induced VH, anxiety, and depression-like behaviors (Ji and Xia, 2022; Ji et al., 2022). The Y-shaped framework integrating sex dimorphism and resilience mechanisms following ELS is illustrated in Figure 2. Such interventions may work by fostering resilience-promoting neuroadaptations. Conversely, the lack of adaptive resilience mechanisms is evident in models showing that neonatal MS disrupts chronic stress coping in males, impairing their ability to habituate to repeated homotypic stress, while females may develop adaptive habituation (Bülbül and Sinen, 2021). Ultimately, understanding the modifiers of risk and trajectory is paramount. It moves the field beyond a deterministic view of ELS toward a nuanced framework that accounts for individual differences and informs the development of targeted therapeutic strategies aimed at enhancing resilience.

## 8 Translational implications and future directions: from mechanistic insights to novel therapeutic strategies

The profound understanding of ELS as a critical etiological factor in VH and DGBIs provides a solid foundation for developing novel, mechanism-based therapeutic strategies. The recognition that ELS is associated with lasting molecular, cellular, and systemic alterations across the microbiota-gut-brain (MGB) axis—as demonstrated in preclinical models—shifts the therapeutic paradigm from mere symptom management toward interventions that may modify the underlying pathophysiology or even promote resilience. A critical roadmap for future research emphasizes the need to integrate components of the brain-gut axis, considering biological sex, genetic background, and early-life experiences to identify biomarkers and endophenotypes that can guide personalized treatment (Chang et al., 2018). Translating preclinical discoveries into clinical applications requires a concerted effort to bridge mechanistic insights with patient-centered care.

Pharmacological interventions are evolving to target specific pathways dysregulated by ELS. The efficacy of the HDAC inhibitor SAHA in reversing ELS-induced VH and anxiety in rodents highlights the therapeutic potential of epigenetic modifiers (Moloney et al., 2015; Melchior et al., 2018). Similarly, targeting specific neurotransmission and signaling pathways holds promise. The β3-adrenoceptor agonist CL-316243 ameliorates VH in maternally separated rats by modulating tryptophan metabolism and enteric neuronal function (Collins et al., 2024). Inhibition of peripheral CGRP signaling with a monoclonal antibody reverses colonic hypersensitivity induced by both adult and ELS (Noor-Mohammadi et al., 2021). Other targeted approaches include enhancing spinal glutamate reuptake with riluzole (Gosselin et al., 2010), inhibiting the RET kinase pathway (Russell et al., 2019), antagonizing CRF1R (Hasegawa et al., 2025), and activating guanylate cyclase-C with linaclotide, which also improves gut barrier function (Ligon et al., 2018). More recent targets include the EphrinB2/EphB2 signaling in the spinal cord (Guo et al., 2025) and the GLP2-Wnt1 axis for epithelial repair (Chen et al., 2024).

Nutritional and microbiota-targeted interventions offer promising, often prophylactic, strategies. Supplementation with MFGM, alone or combined with a prebiotic blend, from weaning onwards can prevent or ameliorate ELS-induced VH, cognitive deficits, and HPA axis dysregulation (Collins et al., 2022; O’Mahony et al., 2020). Probiotic administration, such as *Lactobacillus rhamnosus* GG, can prevent the development of stress hypersensitivity and microbiota alterations (McVey et al., 2020). Furthermore, soluble mediators from probiotics can mimic these beneficial effects, opening avenues for postbiotic therapies (McVey et al., 2020). Dietary components themselves can be protective, as evidenced by a maternal high-fat diet during gestation and lactation preventing the programming effects of concomitant MS (Rincel et al., 2016). Herbal remedies like *S. chinensis* and gallic acid also show efficacy in reversing VH in preclinical models (Yang et al., 2012; Guo et al., 2025).

**FIGURE 3:**
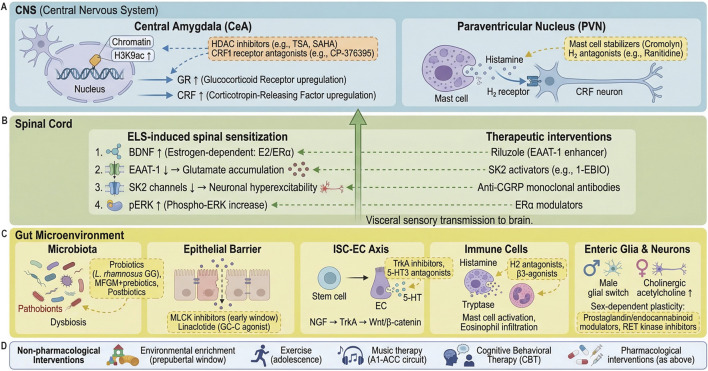
Multilevel mechanisms and therapeutic interventions of early life stress-induced visceral hypersensitivity along the brain-gut axis. This schematic illustrates the pathophysiological mechanisms and corresponding therapeutic strategies for early life stress (ELS)-induced visceral hypersensitivity (VH), organized across three anatomical levels of the brain-gut axis. **(A)** Central nervous system (CNS) level. In the central amygdala (CeA), ELS induces epigenetic upregulation of glucocorticoid receptor (GR) and corticotropin-releasing factor (CRF), characterized by increased histone H3K9 acetylation (H3K9ac). In the paraventricular nucleus (PVN), mast cell-derived histamine activates CRF neurons via H_2_ receptors. These central nodes are targeted by HDAC inhibitors, CRF1R antagonists, mast cell stabilizers (cromolyn), and H_2_ receptor antagonists. **(B)** Spinal cord level. ELS-induced spinal sensitization involves: estrogen-dependent BDNF upregulation, downregulation of EAAT-1 leading to glutamate accumulation, downregulation of SK2 channels causing neuronal hyperexcitability, and increased pERK phosphorylation. Corresponding interventions include riluzole (EAAT-1 enhancer), SK2 activators, anti-CGRP monoclonal antibodies, and ERα modulators. **(C)** Gut microenvironment level. Peripheral pathophysiology is subdivided into five interconnected compartments: (1) Microbiota dysbiosis targeted by probiotics, MFGM + prebiotics, and postbiotics; (2) Epithelial barrier disruption with tight junction impairment; (3) ISC-EC axis showing NGF-TrkA-Wnt-driven enterochromaffin cell hyperplasia and serotonin overproduction, targeted by TrkA inhibitors and 5-HT_3_ antagonists; (4) Immune cells, including mast cell activation and eosinophil infiltration; (5) Enteric glia and neurons exhibiting sex-dependent plasticity (male glial phenotypic switch, female cholinergic upregulation), targeted by prostaglandin/endocannabinoid modulators and RET kinase inhibitors. **(D)** Non-pharmacological interventions. These include environmental enrichment (prepubertal window), exercise (adolescence), music therapy (via A1-ACC circuit), and cognitive behavioral therapy (CBT). Dashed arrows indicate therapeutic targeting relationships. Abbreviations: CNS, Central Nervous System; CeA, Central Amygdala; H3K9ac, Histone H3 Lysine 9 Acetylation; HDAC, Histone Deacetylase; TSA, Trichostatin A; SAHA, Suberoylanilide Hydroxamic Acid; CRF1R, Corticotropin-Releasing Factor Receptor 1; GR, Glucocorticoid Receptor; CRF, Corticotropin-Releasing Factor; PVN, Paraventricular Nucleus; CRF, Corticotropin-Releasing Factor; H_2_, Histamine Receptor Type 2; ELS, Early Life Stress; BDNF, Brain-Derived Neurotrophic Factor; E2, Estradiol; ERα, Estrogen Receptor Alpha; EAAT-1, Excitatory Amino Acid Transporter-1; SK2, Small-Conductance Calcium-Activated Potassium Channel Type 2; pERK, Phosphorylated Extracellular Signal-Regulated Kinase; CGRP, Calcitonin Gene-Related Peptide; MFGM, Milk Fat Globule Membrane; ISC, Intestinal Stem Cell; EC, Enterochromaffin Cell; NGF, Nerve Growth Factor; TrkA, Tropomyosin Receptor Kinase A; 5-HT, 5-Hydroxytryptamine (Serotonin); 5-HT_3_, Serotonin Receptor Type 3; RET, Rearranged During Transfection; A1, Primary Auditory Cortex; ACC, Anterior Cingulate Cortex; CBT, Cognitive Behavioral Therapy. Created with BioRender.com.

Non-pharmacological and lifestyle interventions are cornerstone strategies, particularly given the bidirectional brain-gut dysfunction, and a comprehensive summary of therapeutic interventions targeting ELS-induced VH is presented in Table 2. Behavioral therapies, including cognitive-behavioral therapy (CBT), directly target central pain amplification and maladaptive coping, thereby improving gastrointestinal-specific quality of life (Liu, 2025; Jagielski and Riehl, 2021). Physical exercise during adolescence can mitigate ELS-induced anxiety and associated bowel oxidative stress (Khorjahani et al., 2020). Intriguingly, mechanistic insights are now revealing how such interventions work. For instance, environmental enrichment during prepuberty can prevent ELS-induced phenotypes (Ji and Xia, 2022; Ji et al., 2022), and recent research has delineated a specific neural circuit from the primary auditory cortex to the anterior cingulate cortex that mediates both stress-induced visceral pain and the analgesic effects of music (Yu et al., 2026). An integrated summary of mechanisms and therapeutic interventions for stress-related visceral pain is presented in Figure 3.

**TABLE 2:** Therapeutic interventions targeting ELS-induced visceral hypersensitivity.

Target/pathway	Intervention	Site of action	Timing	Primary effects	Sex dependency	Evidence level	Clinical translation potential	References
Epigenetic Modification	HDAC inhibitors (TSA)/HAT inhibitor (Garcinol)	CNS (CeA)	Adult	• Attenuates VH • Modifies H3K9 acetylation and GR binding at CRF promoter	Female-specific (Unpredictable ELS model)	Strong (Direct evidence in ELS models)	• HDACi approved (oncology) • Requires targeted CNS delivery	Meerveld and Johnson (2018), Louwies et al. (2023), Louwies and Greenwood-Van (2020)
CRF Signaling	CRF1R antagonists (CP-376395)	CNS (CeA)	Adult	• Significantly decreases VH	Female-specific (Unpredictable ELS model)	Strong (Direct evidence in ELS models)	• In clinical development for other indications • Previously tested for anxiety	Hasegawa et al. (2025), Prusator and Greenwood-Van (2017)
Glutamate Transport	Riluzole (EAAT enhancer)	Spinal Cord	Adult	• Normalizes MS-induced VH • Increases glutamate reuptake	Not specified	Strong (Direct evidence in MS model)	• Approved (ALS) • Rapidly translatable	O’Mahony et al. (2011), Gosselin et al. (2010), Moloney et al. (2016)
CGRP Signaling	Anti-CGRP F (ab’)_2_ antibody	Peripheral/Spinal Cord	Adult	• Attenuates stress-induced colonic hypersensitivity • Inhibits spinal pERK expression	Not specified	Moderate (Evidence in adult stress and ELS models)	• Approved (migraine) • High translational potential	Noor-Mohammadi et al. (2021)
5-HT Signaling	5-HT_3_ antagonists/5-HT_4_ agonists	Gut/Spinal Cord	Adult	• Modulates pain and motility (mentioned in context of IBS)	Female-predominant (implied for IBS)	Indirect (Discussed in review/context, not direct ELS intervention data)	• Approved (antiemetic/prokinetic) • Clinical efficacy in IBS debated	Accarie and Vanuytsel (2020), Collins et al. (2024)
β3-Adrenoceptor	β3-agonist (CL-316243)	Enteric Neurons/Peripheral & Central	Adult	• Ameliorates MS-induced VH • Modulates tryptophan metabolism and secretomotor activity	Not specified	Strong (Direct evidence in MS model)	• Preclinical • Novel mechanism for gut-brain axis	Collins et al. (2024)
SK2 Channels	SK2 activator (1-EBIO)	Spinal Cord (Dorsal Horn)	Adult	• Alleviates MS-induced VH • Reverses neuronal hyperexcitability	Not specified i	Strong (Direct evidence in MS model)	• Preclinical • Novel target	Wu et al. (2020)
Mast Cells	Mast cell stabilizer (Cromolyn)	CNS (PVN)	Adult	• Represses CRF neuronal activation induced by MS	Not specified	Moderate (Direct evidence in ELS model)	• Approved (allergy) • Drug repurposing potential	Chen et al. (2021b), Chen et al. (2023)
Microbiota	Probiotics/Prebiotics/Postbiotics	Gut Microbiota	Developmental/Adult	• Attenuates VH, improves barrier, modulates HPA axis (theoretical/indirect mention)	Both sexes	Indirect (Mentioned in review context, no direct ELS intervention data)	• Commercialized • High safety profile	Rincel and Darnaudéry (2020)
Gut Barrier	GC-C agonist (Linaclotide)	Intestinal Epithelium	Adult	• Attenuates ELS-induced colonic and bladder hypersensitivity • Reverses barrier dysfunction	Female-specific (Validated in female rats)	Strong (Direct evidence in ELS model)	• Approved (IBS-C) • High translational potential	Ligon et al. (2018)
Environmental enrichment	Enriched environment housing	CNS/behavior	Adolescence (Developmental window)	• Reverses VH, anxiety/depression, promotes resilience	Both sexes	Strong	• Behavioral intervention • Clinically translatable concept	Ji and Xia (2022), Ji et al. (2022)
Histamine signaling	H_2_ receptor antagonist (Ranitidine)	CNS (PVN)	Adult	• Alleviates NMS-induced adult visceral pain • Inhibits CRF neuronal activation in PVN • Inhibits PKA-CREB signaling phosphorylation	Study used male mice (to exclude estrogen effects)	Strong (Provides direct pharmacological intervention evidence in ELS-NMS model)	• Approved drug (gastric acid) • High repurposing potential, but requires validation of central delivery route	Chen et al. (2023)

Abbreviations: 5-HT, serotonin; ACC, anterior cingulate cortex; A1, primary auditory cortex; ALS, amyotrophic lateral sclerosis; CeA, central nucleus of the amygdala; CGRP, Calcitonin gene-related peptide; CNS, central nervous system; CRF, Corticotropin-releasing factor; EAAT, excitatory amino acid transporter; ELS, early life stress; ERK, Extracellular signal-regulated kinase; GC-C, Guanylate cyclase-C; GR, glucocorticoid receptor; H2, Histamine receptor type 2; H3K9, Histone three lysine 9; HAT, histone acetyltransferase; HDAC, histone deacetylase; HDACi, Histone deacetylase inhibitor; HPA, Hypothalamic-pituitary-adrenal axis; IBS, irritable bowel syndrome; IBS-C, irritable bowel syndrome with constipation; MFGM, milk fat globule membrane; MLCK, myosin light chain kinase; MS, maternal separation; NCI, neonatal colon inflammation; pERK, Phosphorylated extracellular signal-regulated kinase; PVN, paraventricular nucleus of the hypothalamus; SK2, Small conductance calcium-activated potassium channel type 2; TSA, Trichostatin A; VH, visceral hypersensitivity.

The future of DGBI management lies in integrated, multidisciplinary care models that address the full complexity of the brain-gut axis. Clinical evidence shows that patients with DGBIs, who frequently have histories of ACEs and comorbid anxiety or depression, benefit significantly from psychiatric care integrated within a gastroenterology setting (Dendrinos et al., 2025). This holistic model aligns with the pathophysiological understanding that ELS affects both top-down and bottom-up pathways. Effective treatment will likely require combination strategies (Talley et al., 2015). Furthermore, the concept of restoring barrier function as a therapeutic target is gaining traction, as pharmacological inhibition of myosin light chain kinase in neonates during the stress period can prevent long-term consequences of ELS (Rincel et al., 2019).

Critical challenges and future directions remain. First, improving the translational value of animal models is essential, requiring models that better capture the heterogeneity of human DGBIs (Accarie and Vanuytsel, 2020). Second, there is an urgent need to identify biomarkers that can predict individual treatment response. A *post hoc* analysis suggested that a history of childhood abuse did not predict response to paroxetine in IBS, but larger studies are needed (Han et al., 2009). Third, understanding and improving treatment adherence is crucial, as objective adherence to short-course therapies like rifaximin can be poor (Sherwin et al., 2020). Finally, the field must prioritize prevention and early intervention strategies. The enduring effects of ELS, while profound, are not immutable. Interventions during critical developmental windows demonstrate the potential to block or build resilience against the programming effects of early adversity (O’Mahony et al., 2020; Melchior et al., 2018). Advancing these strategies from preclinical validation to clinical application will require robust longitudinal studies and a commitment to personalized medicine.

The recognition of ELS as a critical etiological factor in DGBIs presents a unique opportunity to move beyond the traditional “one-size-fits-all” symptomatic management. However, the remarkable clinical heterogeneity among patients—whereby some present with predominantly peripheral symptoms (e.g., diarrhea, bloating, food intolerance) while others exhibit a more central phenotype (e.g., hypervigilance, anxiety, stress-sensitivity)—underscores the urgent need for a precision medicine approach. A major challenge for the field is to translate the intricate multi-level mechanisms outlined in this review into a clinically actionable framework that can stratify patients based on their dominant underlying pathophysiology. Table 3 proposes a novel endotype-based classification system for ELS-associated DGBIs, integrating candidate biomarkers with proposed dominant mechanisms and personalized treatment strategies. This framework synthesizes evidence from the preclinical and clinical literature reviewed above, aiming to guide future clinical trial design and, ultimately, bedside decision-making.

**TABLE 3:** Integrating biomarkers, dominant mechanisms, and personalized treatment strategies for ELS-associated DGBIs.

Patient endotype	Proposed biomarkers	Core pathophysiology	Personalized treatment strategy	Expected clinical benefit	References
Type A: peripheral-dominant (immune-barrier)	• Mast cell tryptase in colonic biopsies ↑ • Intestinal permeability (lactulose/mannitol test) ↑ • Fecal eosinophil-derived mediators ↑ • Gut dysbiosis (e.g., reduced Lactobacillus)	• Mast cell and eosinophil activation • Tight junction disruption • Dysbiosis • EC cell hyperplasia and 5-HT overproduction	1. Mast cell stabilizers (Cromolyn) 2. H2 receptor antagonists (Ranitidine) 3. Probiotics/Postbiotics (L. rhamnosus GG) 4. Gut barrier protectants (Linaclotide)	• Reduced peripheral sensitization • Normalized barrier function • Improved stool consistency	Riba et al. (2018), Chen Z. et al. (2021), Chen et al. (2023), Chen et al. (2021c), Wu et al. (2020), Duan et al. (2026), McClain et al. (2020), Rincel and Darnaudéry (2020), O’Mahony et al. (2020), Ligon et al. (2018), Rincel et al. (2019)
Type B: central-dominant (stress-circuitry)	• Salivary cortisol (altered diurnal rhythm) ↑ • Epigenetic marks (e.g., NR3C1 promoter methylation, H3K9ac in blood)	• CRF system hyperactivity (CeA, PVN) • Spinal sensitization (BDNF, SK2 downregulation) • Epigenetic reprogramming (GR/CRF)	1. CRF1R antagonists 2. HDAC inhibitors (TSA) 3. EAAT enhancers (Riluzole) 4. SK2 activators (1-EBIO)	• Reduced stress-induced flares • Normalized central pain processing • Improved anxiety and depression	Meerveld and Johnson (2018), Hasegawa et al. (2025), Prusator and Greenwood-Van (2017), Gosselin et al. (2010), Wu et al. (2020), O’Malley et al. (2011), Louwies et al. (2023), Louwies and Greenwood-Van (2020), Sugiyama and Shiotani (2021)
Type C: mixed/overlap type	• Biomarkers for both Type A and B present	• Synergistic activation of both peripheral immune and central stress systems • Likely represents a “two-hit” scenario	Combination strategy 1. Central (e.g., CRF1R antagonist) 2. Peripheral (e.g., Mast cell stabilizer or Probiotics)	• Most impactful, but also most complex • Requires careful trialing	Winston et al. (2014), Louwies et al. (2023)

Abbreviations: 5-HT: serotonin; ACC: anterior cingulate cortex; BDNF: Brain-derived neurotrophic factor; CBT: cognitive behavioral therapy; CeA: central nucleus of the amygdala; CRF: Corticotropin-releasing factor; CRF1R: Corticotropin-releasing factor receptor type 1; DGBI: Disorder of gut-brain interaction; EAAT: excitatory amino acid transporter; EC: enterochromaffin cell; EDN: Eosinophil-derived neurotoxin; ELS: early life stress; H_2_: Histamine receptor type 2; H3K9ac: Histone three lysine nine acetylation; HDAC: histone deacetylase; IgG: Immunoglobulin G; MBSR: Mindfulness-based stress reduction; MRI: magnetic resonance imaging; PFC: prefrontal cortex; SK2: Small conductance calcium-activated potassium channel type 2; TSA: Trichostatin A.

This stratification model is based on the concept that the specific nature of ELS (e.g., predictability), the timing of subsequent “second hits,” and sex-specific biological factors all contribute to a unique pathological signature in each patient (Winston et al., 2014; Louwies et al., 2023). For instance, a patient presenting with ELS history, prominent bloating, diarrhea, and evidence of mast cell activation on biopsy could be classified as Type A (Peripheral-Dominant). In this scenario, a trial of a peripherally-acting agent such as the mast cell stabilizer cromolyn or the guanylate cyclase-C agonist linaclotide, which has shown efficacy in a female ELS model (Ligon et al., 2018), would be a rational first-line approach. Conversely, a patient whose symptoms are tightly coupled with psychosocial stress, and who displays marked anxiety and hypervigilance, may represent a Type B (Central-Dominant) endotype, for whom interventions targeting central stress circuitry, such as CRF1R antagonists (Prusator and Greenwood-Van, 2017) or HDAC inhibitors (Louwies et al., 2023; Melchior et al., 2018), or evidence-based behavioral therapies like CBT (Dendrinos et al., 2025; Jagielski and Riehl, 2021), may be more effective. The Mixed Type (Type C) likely accounts for the most severely affected individuals, often with a “two-hit” history (Winston et al., 2014; Louwies et al., 2023), for whom a sequential, multimodal therapeutic strategy will be essential. This framework is inherently hypothesis-generating. A fundamental next step for the field is to prospectively validate these candidate biomarkers and their association with treatment response. By explicitly linking the multi-level mechanisms of ELS-induced VH to testable clinical hypotheses, this endotype-based approach offers a clear path toward the ultimate goal of precision medicine for this complex patient population.

## 9 Conclusion and perspectives

The intricate and enduring relationship between ELS and the subsequent development of VH stands as a cornerstone in the pathophysiology of DGBIs. This review has synthesized a substantial body of evidence, demonstrating that adverse experiences during critical developmental windows can program a lasting vulnerability characterized by heightened pain perception, emotional dysregulation, and altered gut homeostasis. Epidemiological studies consistently link various forms of ELS—including abuse, neglect, parental loss, and severe early medical interventions—to a significantly increased risk of DGBIs like IBS in later life (Burke et al., 2017; Low et al., 2020; Rey and Talley, 2009; Fritz et al., 2025; Jang et al., 2022). Preclinical models have been instrumental in recapitulating this phenomenon, revealing that ELS, such as MS, reliably induces VH and anxiety-like behaviors in adulthood, providing a tractable system for mechanistic dissection (Fuentes and Christianson, 2018; Accarie and Vanuytsel, 2020; Sugiyama and Shiotani, 2021).

The pathophysiology emerges not from a single linear pathway but from the dysregulated interplay of multiple, interconnected systems along the brain-gut axis. Central to this is a maladaptive stress response. It often involves persistent alterations in the HPA axis and enhanced CRF signaling within limbic circuits like the amygdala. Evidence suggests this may amplify central pain processing (Burke et al., 2017; Meerveld and Johnson, 2018; Buckley et al., 2014). These central changes are paralleled and reinforced by peripheral perturbations. ELS can compromise the intestinal epithelial barrier, prime mucosal immune cells (e.g., mast cells and macrophages) for hyper-reactivity, and alter the ENS’s neurochemical coding, creating a state of peripheral sensitization (Buckley et al., 2014; Sugiyama and Shiotani, 2021). Furthermore, ELS-induced shifts in the gut microbiota composition and function contribute to this pro-nociceptive milieu, influencing immune tone and gut-brain communication (Zhou et al., 2023; O’Mahony et al., 2020; Dinan et al., 2010). Epigenetic mechanisms critically serve as the molecular interface that translates transient environmental exposures into stable phenotypic changes, mediating the long-term effects on gene expression in both the brain and the gut (Liu et al., 2017; Meerveld and Johnson, 2018; Théodorou, 2013; Dinan et al., 2010).

This comprehensive model underscores the validity of the biopsychosocial framework for understanding DGBIs. In this framework, biological vulnerability—shaped by early experience—interacts with psychological states and social context. Together, they determine symptom onset, severity, and healthcare-seeking behavior (Almounajed and Drossman, 1996; Levy et al., 2006; Bonilla and Saps, 2013; Barad and Saps, 2008). Psychological comorbidities, particularly anxiety and depression, are not merely consequences but integral components of this dysregulated axis, both contributing to and being exacerbated by visceral distress (Woods et al., 2026; Liu, 2025; Berens et al., 2019; Lee et al., 2020). The association between higher ACE scores and DGBI diagnoses, often mediated by anxiety, highlights the clinical importance of assessing psychosocial history (Dendrinos et al., 2025; Fritz et al., 2025; Lee et al., 2020).

Significant progress has been made, yet critical knowledge gaps and challenges persist, dictating essential future directions. First, enhancing the translational relevance of preclinical research is paramount. While animal models have been invaluable, developing paradigms that better capture the heterogeneity of human DGBIs—including sex differences in stress susceptibility and resilience (Bülbül and Sinen, 2021)—and the “multiple-hit” hypothesis (e.g., ELS combined with later infection or dietary stress) will yield more clinically predictive insights (Accarie and Vanuytsel, 2020; Hasegawa et al., 2025). Second, the field urgently needs validated biomarkers to stratify patients. Moving beyond symptom-based diagnoses to identify molecular, microbial, or neuroimaging signatures associated with specific ELS-related endophenotypes, including factors like illness identity and visceral sensitivity (Thomann et al., 2023), could revolutionize personalized treatment (Chang et al., 2018). Third, improving real-world treatment outcomes requires a deeper understanding of factors influencing adherence, as even short-course therapies can suffer from poor objective adherence despite significant patient burden (Sherwin et al., 2020). Fourth, and most importantly, research must increasingly pivot towards prevention and early intervention. The evidence that ELS effects are “programmed” but not immutable is a source of therapeutic optimism. However, it is important to distinguish between near-term clinically feasible interventions and longer-term exploratory strategies. Near-term clinically feasible interventions include: (1) routine screening for ACEs in clinical settings (Dendrinos et al., 2025; Heitkemper et al., 2011); evidence-based behavioral therapies such as CBT and gut-directed hypnosis (Woods et al., 2026; Liu, 2025; Jagielski and Riehl, 2021); (3) promotion of exclusive breastfeeding, which has been shown to be a protective factor against early DGBIs (Chanpong et al., 2025); and (4) nutritional approaches with established safety profiles, such as probiotic supplementation (McVey et al., 2020; Barouei et al., 2009) and MFGM combined with prebiotics (O’Mahony et al., 2020). Exploratory or longer-term strategies, which remain largely experimental and require further validation before clinical translation, include: (1) pharmacological targeting of epigenetic writers/erasers (e.g., HDAC inhibitors) (Louwies et al., 2023; Moloney et al., 2015; Melchior et al., 2018); (2) oxytocin signaling pathways (Melchior et al., 2018); and (3) other novel molecular targets identified in preclinical models. These approaches hold immense promise for building resilience or mitigating risk, but their safety, efficacy, and feasibility in human populations must be established through rigorous clinical trials.

Ultimately, managing DGBIs rooted in early adversity demands an integrated, multidisciplinary approach. Effective care moves beyond siloed gastroenterological or psychiatric treatment to embrace a holistic model. This includes routine screening for ACEs in clinical settings (Dendrinos et al., 2025; Heitkemper et al., 2011), leveraging effective behavioral therapies like cognitive behavioral therapy and gut-directed hypnosis (Woods et al., 2026; Liu, 2025; Jagielski and Riehl, 2021), and considering psychotropic medications when appropriate (Liu, 2025; Wu, 2012). The convergence of pathways elucidated here argues against a dualistic “mind versus gut” perspective and for a unified systems biology view. Future success hinges on longitudinal birth cohort studies with deep phenotyping and biospecimen collection (Low et al., 2020; Chang et al., 2018), alongside continued investment in basic science to unravel the precise circuits and molecular cascades. These insights chart a course from symptom relief to lifelong prevention for those affected by early-life adversity.
